# Evaluating the influence of music at different sound pressure levels on medical students’ performance of standardized laparoscopic box training exercises

**DOI:** 10.1186/s12909-021-02627-6

**Published:** 2021-04-13

**Authors:** Lisa Katharina Nees, Philipp Grozinger, Natalie Orthmann, Thomas Maximilian Deutsch, André Hennigs, Christoph Domschke, Markus Wallwiener, Joachim Rom, Fabian Riedel

**Affiliations:** 1 Department of Gynecology and Obstetrics, Heidelberg University Hospital, Im Neuenheimer Feld 440, D-69120 Heidelberg, Germany; 2 Asklepios Klinik Hamburg-Altona, Department of Gynecology and Obstetric, Hamburg, Germany; 3 Klinikum Frankfurt-Hoechst, Department of Gynecology and Obstetrics, Frankfurt, Germany

**Keywords:** Laparoscopy, Box training, Simulation-based training, Music exposure, Sound pressure level

## Abstract

**Background:**

The influence of music on the performance of surgical procedures such as laparoscopy is controversial and methodologically difficult to quantify. Here, outcome measurements using laparoscopic box training tools under standardized conditions might offer a feasible approach. To date, the effect of music exposure at different sound pressure levels (SPL) on outcome has not been evaluated systematically for laparoscopic novices.

**Methods:**

Between May 2017 and October 2018, *n* = 87 students (49 males, 38 females) from Heidelberg University Medical School performed three different laparoscopy exercises using the “Luebecker Toolbox” that were repeated twice under standardized conditions. Time was recorded for each run. All students were randomly assigned to four groups exposed to the same music compilation but at different SPLs (50–80 dB), an acoustically shielded (earplug) group, or a control group (no intervention).

**Results:**

Best absolute performance was shown under exposure to 70 dB in all three exercises (a, b, c) with mean performance time of 121, 142, and 115 s (*p* < 0.05 for a and c). For the control group mean performance times were 157, 144, and 150 s, respectively. In the earplug group, no significant difference in performance was found compared to the control group (*p* > 0.05) except for exercise (a) (*p* = 0.011).

**Conclusion:**

Music exposure seems to have beneficial effects on training performance. In comparison to the control group, significantly better results were reached at 70 dB SPL, while exposure to lower (50 or 60 dB) or higher (80 dB) SPL as well as under acoustic shielding did not influence performance.

## Background

Laparoscopy represents the standard approach for many gynecologic surgery procedures because of its advantages over conventional laparotomy [1]. However, one major factor influencing outcome and efficiency in laparoscopic surgery is training, especially for novices before performing surgeries autonomously [2].

In contrast to a sheltered training setting, there are many distractors that affect novices in particular in the actual surroundings of an operating room (OR) and raise stress levels during surgery. One of them might be exposure to music played in the OR, which affects beginners more than experienced surgeons [3]. In many hospitals, it is common practice to play music in the OR during surgical procedures [4, 5]. Findings have shown that music might affect cognitive performance [6], but limited data is known about its influence on surgical performance. The influence of acoustic factors such as music on laparoscopic technique performance is controversial and methodologically difficult to quantify [7]. Here, outcome measurements performed by surgical novices using laparoscopic box training tools under standardized conditions might offer a feasible approach to answer this question.

Earlier studies of laparoscopic training show that the learning curve is prolonged for novice and intermediate surgeons [8] and that different learning curves exist, depending on the level of laparoscopic training [9]. There is a consensus that educational activities should be intensified and an assessment of surgeons’ skills could be introduced in order to ensure that the quality of treatment is adequate, especially for training residents in gynecology [10].

Here, simulators are accepted as an important means of training and as an objective assessment of psychomotor performance. Standardized tasks can be practiced repeatedly, and simulators provide unbiased and objective measurement of surgery performance. Thus, simulation-based training has become increasingly relevant in laparoscopic gynecologic surgery training. In this context, both box trainers and virtual reality simulators seem to be equally effective as a means of teaching laparoscopic skills to novice learners before entering the OR [11].

In this study we applied standardized exercises on box trainers for evaluating the performance of laparoscopic novices who were exposed to music at different sound pressure levels. To our knowledge, this effect on outcome has not yet been evaluated systematically.

## Methods

### Participants and study design

For the study we recruited students at Heidelberg University Medical School at the end of their clinical curriculum who were participating in a 4-week module in Obstetrics and Gynecology at our hospital [12]. During these modules we offered a voluntary 90-min laparoscopic training course at our in-house skills lab. All module participants were eligible. The number of participants of each training course varied among the dates offered, with a minimum of 4 to a maximum of 8 participants at the same time accompanied by one tutor. As a training tool we used the commercially available “Luebecker Toolbox” (LTB Ltd., Luebeck, Germany; http://www.luebeck-toolbox.com). This system consists of a laparoscopy training box including an integrated camera (connected to a monitor), four standardized modules with the possibility to perform six different exercises, as well as associated didactic videos that are available online [13]. We chose three exercises for the purpose of our study: (a) “Pack Your Luggage” (PYL), (b) “Chinese Jump Rope” (CJR), and (c) “Weaving” (WEA). A detailed description of the exercises has already been published by Laupert et al. [13, 14]. These three exercises offer specific training in instrument handling, hand-eye coordination, and bimanual and crossing instrument use. All participants were given a short oral introduction on general aspects of gynecologic laparoscopic surgery and viewed instructional videos once for each exercise in order to standardize the training procedure. For those students with a dominant left hand, tasks were performed in the opposite direction. Original grasping forceps were used to perform the tasks (Karl Storz SE & Co. KG, Tuttlingen, Germany). All three exercises were performed in the same order and each exercise was repeated three times in a row. There was no training session before. Performance was evaluated by measuring the time needed to complete the exercise. For the purpose of this study all students were randomly assigned to different groups (at the time of the student’s registration for the course), either to one of the intervention groups who were exposed to the same music compilation (“Deep House Autumn Mix 2017- The Best Of Vocal Deep House Nu Disco Music”, found on “YouTube”, uploaded Oct 08th 2017) but at different sound pressure levels (SPL), i.e., exposure of 50 dB vs. 60 dB vs. 70 dB vs. 80 dB (dB), or the noise-shielded group with no music exposure and using conventional foam earplugs (ISO 4869). This additional intervention group was set up to exclude the effect of surrounding noise in comparison to the control group. In the control group the participants performed all exercises under exposure of regular surrounding noise (i.e. talking, noise deriving from instrument handling, etc.). The choice of the music compilation was made as this type of music is often chosen by surgeons at our institutions through its monotonic rhythm and the reserved use of vocal parts. The study was conducted under standardized conditions, i.e. music exposure came from one standardized source (SoundLink Mini Bluetooth Speaker; Bose, Germany) in the middle of a closed rectangle room that was 9 × 3.5 m in diameter. All participants had the same distance to the sound source of around one meter. Respective SPLs for each run were measured constantly with a calibrated sound pressure meter (WT1357, Akozon Ltd., P.R. China) and protocolled regularly every minute during each exercise. Discrepancies in sound pressure levels were adjusted immediately by the tutor. After finishing the exercises, the participants were asked to complete a short survey on statistical factors that might be related to the performance in this study (e.g., handedness, former experience in laparoscopic surgery during an internship, etc.) as well as to evaluate the exercise.

The study was designed as a prospective trial and approved by the Heidelberg University Medical School ethics committee (Register No. S618/2017). All study participants provided written informed consent. Course and study participation were voluntary. Participants were assigned to one of the study groups randomly during the online registration period for the laparoscopy training course according to the time of registration on the different dates offered throughout the semester. All data were collected prospectively and handled anonymously for statistical analyses.

### Statistical analyses

Collected data were transferred to a database in Excel (Microsoft Corporation; Redmond, USA). The statistical analysis was carried out using SPSS® (SPSS Inc., IBM Corporation; Chicago, Illinois, USA). All values given are mean values, ranges, and standard deviation. The significance test was carried out using the T-test to compare the mean values in an independent sample. A *p*-value < 0.05 was considered statistically significant.

## Results

### Study participants

In total, *n* = 87 students took part in our study, 38 females (43.7%) and 49 males (56.3%). All participants were undergraduate students at Heidelberg University Medical School, most of them in the last clinical semester (9th clinical semester: 51%), i.e., at the end of their 5th year. Most of them did not have any practical experience in laparoscopic surgery (67.8%). Around 15% were left-handed, 85% right-handed. Between study groups no significant differences were detected concerning age, gender, handedness, and previous laparoscopy training experience. Table 1 shows detailed characteristics.

**Table 1:** Course participants characteristics (*n* = 87) [absolute and percent]

Participant characteristics (***n*** = 87)		
	Number	[%]
**Gender**		
female	38	43.7
male	49	56.3
**Handedness**		
right	74	85.1
left	13	14.9
**Semester**		
< 9th	15	17.2
9th	44	50.6
10th	15	17.2
> 10th	13	15.0
**Overall previous laparoscopy training experience (hours)**		
none	59	67.8
≤ 5	16	18.4
6–10	7	8.0
> 10	5	5.7

### Performance under music exposure at different SPL compared to the control group

**Fig. 1:**
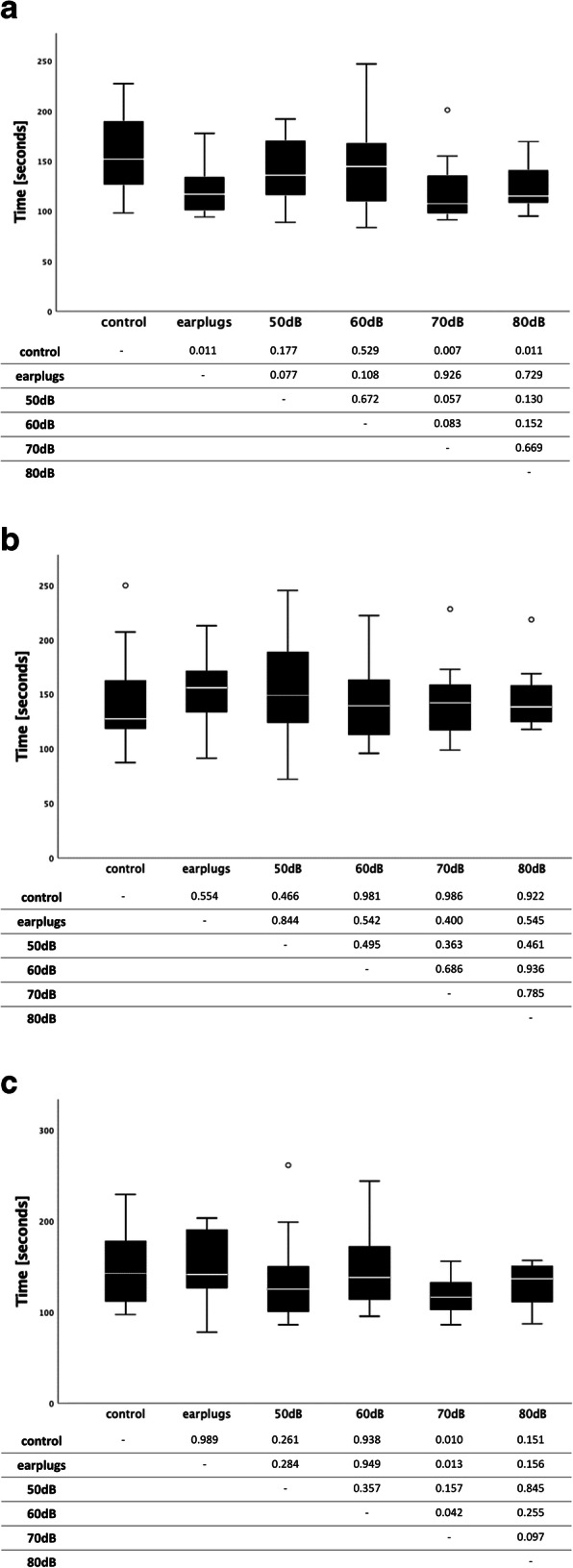
Performance of the three laparoscopic exercises for the control group and five intervention groups (earplug, SPL 50–80 dB). **a**. Exercise (a), “Pack your luggage”; mean values of the required times are shown on the ordinate with their 25 and 75% quartiles against the respective groups on the abscissa; *p*-values for the comparisons between each group are shown below; ° = outlier. **b**. Exercise (b), “Chinese Jump Rope”. Mean values of the required times are shown on the ordinate with their 25 and 75% quartiles against the respective groups on the abscissa; *p*-values for the comparisons between each group are shown below; ° = outlier. **c**. Exercise (c), “Weaving”. Mean values of the required times are shown on the ordinate with their 25 and 75% quartiles against the respective groups on the abscissa; *p*-values for the comparisons between each group are shown below; ° = outlier

In exercise (a) best absolute mean performance was measured under exposure at 70 dB (121 s), which was significantly better than the control group (157 s; *p* = 0.007). Furthermore, the groups exposed to music at 80 dB (125 s) and the sound-shielded group (122 s) were significantly faster than the control group (*p* = 0.011). For exercise (b), no significant difference in performance between the groups was found, although the 70-dB group performed best (142 s). In exercise (c), the 70-dB group (115 s) again performed significantly better than the control group (150 s; *p* = 0.010). All other interventional groups showed no significant differences compared to the control group. Table 2 and Figs. 1a-c present detailed results for the three different laparoscopic exercises.

**Table 2:** Number of participants and performance for exercises a, b, and c in [s] (mean ± SD)

Group	Number of participants	Performance exercise a [s] mean (± SD)	Performance exercise b [s] mean (± SD)	Performance exercise c [s] mean (± SD)
**Control group**	16	157 (± 38.5)	144 (± 45.0)	150 (± 44.3)
**Earplugs**	12	122 (± 26.9)	153 (± 34.0)	150 (± 38.2)
**50 dB**	20	142 (± 31.1)	156 (± 48.9)	133 (± 43.5)
**60 dB**	12	147 (± 45.7)	144 (± 36.7)	148 (± 47.9)
**70 dB**	15	121 (± 31.0)	142 (± 33.7)	115 (± 20.0)
**80 dB**	12	125 (± 23.5)	145 (± 28.5)	130 (± 24.7)
**Total**	87

### Relative performance improvements between first and third run

**Fig. 2:**
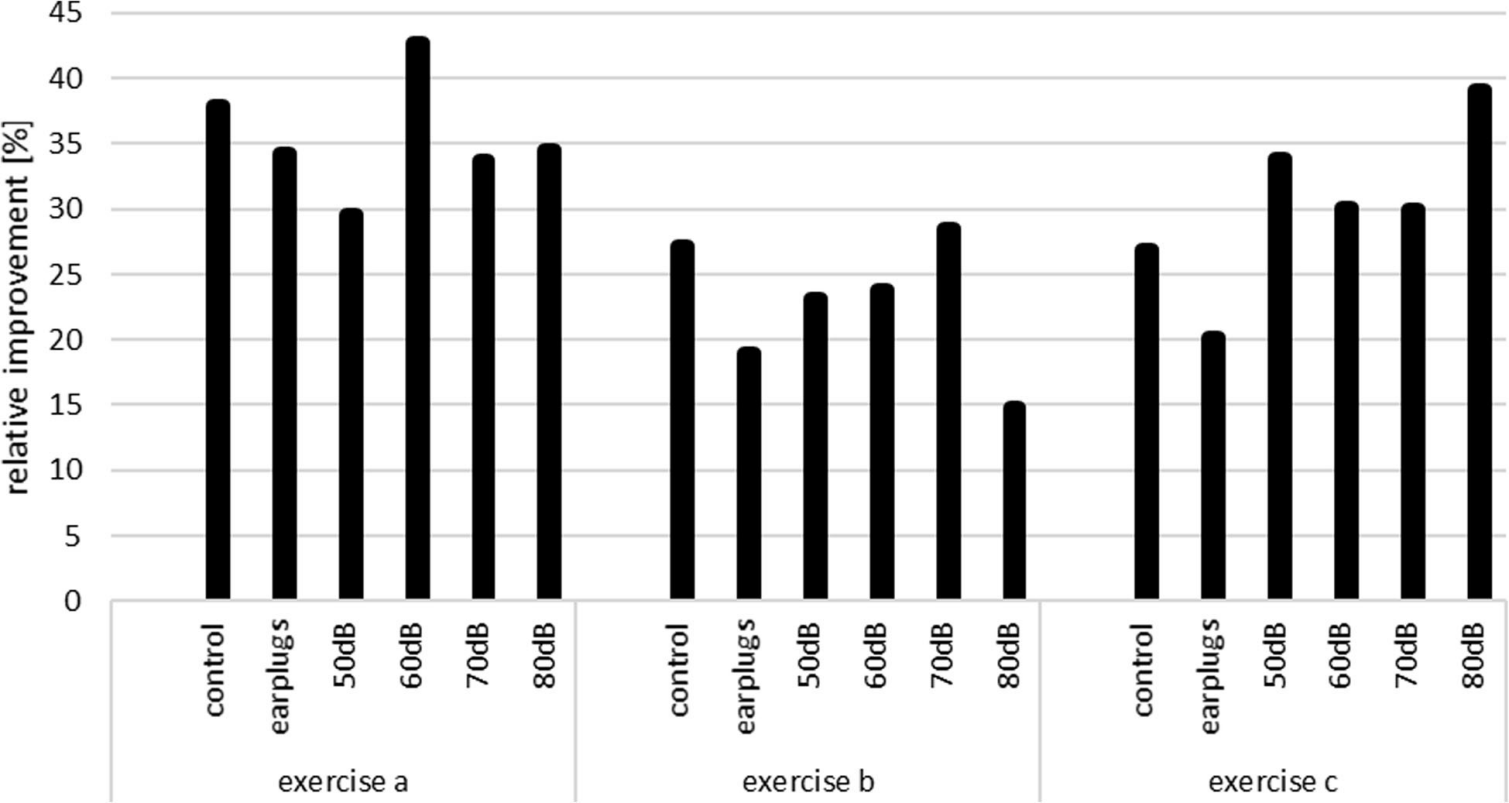
Relative performance improvements between first and third run separated for exercises a, b, and c and different sound pressure levels plus control group / earplug group (in %)

**Fig. 3:**
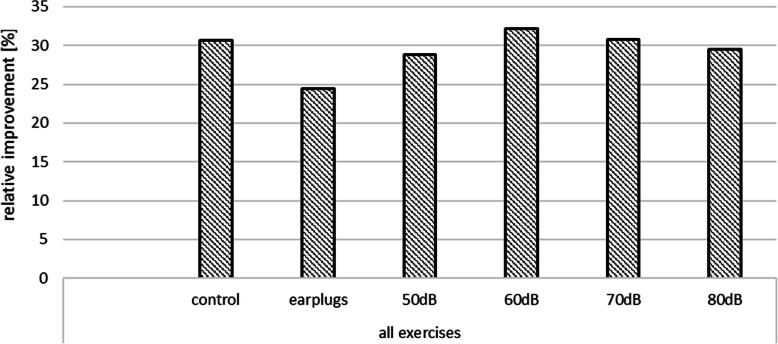
Relative overall performance improvements (combined for all exercises a, b, and c) between first and third run for different sound pressure levels plus control group / earplug group (in %)

The relative improvements seen in the third run in comparison to the first run for all exercises are shown in Fig. 2. The highest relative improvements are seen for exercise (a) at 60 dB (42.7%), for exercise (b) at 70 dB (28.5%), and for exercise (c) at 80 dB (39.1%). However, the t-test for the independent sample does not show statistical significance (*p* = 0.05). Furthermore, overall improvements for the different SPLs for all exercises between the first and third run were calculated. The highest relative improvements are seen at 60 dB with 32.2%. Detailed results are presented in Fig. 3.

### Subjective music perception

Concerning the post-hoc perception, 81.4% of all participants in the interventional groups did not feel distracted by music during the exercises, revealing a significant difference between the groups (50 dB: 0% vs. 60 dB: 41.6% vs. 70 dB: 0% vs. 80 dB: 50.0%). Moreover, overall course satisfaction was high (9.2 out of 10.0 points).

## Discussion

The positive effect of music in motivating people has already been studied in a wide variety of areas of life, especially in physical leisure activities [15]. In therapeutic settings, too, the beneficial effects of music are used, for example, for pain management [16] or supportive therapy in cancer patients [17]. From the perspective of the occupational setting, music is also played at work: In many hospitals, it is common practice to play music in the OR during surgical procedures [4, 5]. Here, music is being played in the background for the purpose of entertaining surgical staff, which must be distinguished from music being played in the preoperative setting to reduce anxiety among patients before surgery [18]. The decision as to whether music will be played in the OR or the kind of music that is chosen is predominantly the privilege of the senior surgeon [19].

The effect of music in this specific occupational setting is difficult to quantify. For other, nonhospital occupational settings, a study showed that background music is likely to reduce worker attention and performance [20]. For the OR setting, findings have shown that music is one of several mental distractors that might influence surgical performance negatively, but results differ [21, 22]. A recent meta-analysis stated that the evidence to definitively determine whether music has a beneficial effect on surgical performance in a simulated setting is not sufficient [23]. When analyzing the effect of music in the OR, music is often just one factor among others comprising the general background noise there. The amplitude of background noise, in turn, depends on the specialty; e.g., an obstetrics OR has a comparably high baseline noise level [24].

Nonetheless, it is difficult to quantify the effect of music on surgical outcome. This is due to the varying test persons (advanced surgeons vs. beginners), different music genres and SPLs, as well as differing complexity of the tasks to be performed [7]. Finally, there might be a difference between measurement under standardized training conditions (with usage of simulators) or in the actual environment of an OR. In our study, we used a standardized training setting for laparoscopic exercises that were performed by surgical novices in order to control the relevant influencing factors. Laparoscopy is an adequate tool because it combines manual and neurocognitive requirements. The effect of noise (in general) on laparoscopic performance specifically is controversial. For experienced surgeons, one study showed that background noise at 113 dB had a negative impact on surgical laparoscopic performance [25], whereas another study on the effect of noise at 80–85 dB and background music showed no difference in task performance in terms of the time taken to complete a task [26]. Here, one must keep in mind that those noise levels are higher than most recommended standards for an occupational environment [27]. The SPLs used in the two studies also differed greatly, which makes a comparison difficult, but as an explanation it was assumed that experienced surgeons can effectively “block out” noise and music on a higher SPL of 80–85 dB. This is probably due to the high levels of concentration required to perform a complex surgical task. Recent studies of abdominal surgeries showed that surgeons’ concentration was not impaired by measured noise levels [28] and there were hints that music might even reduce the heart rate, blood pressure, and muscle effort of surgeons while at the same time increasing the accuracy of surgical tasks [29].

In this context, the effect of routine and training in manual tasks seems to play an important role: Especially younger surgeons (i.e., interns or residents) seem more likely to be distracted by disturbing factors in the OR [30, 31], not only by music but also telephone calls [32]. Under distracting conditions, the medical interns showed a significant decline in task performance (overall task score, task errors, and operating time) and significantly increased levels of irritation toward both the assistant handling the laparoscope in a nonoptimal way and the sources of social distraction.

Due to the fact that the influence of music on performance outcome of laparoscopic techniques in a real-life setting is controversial and methodologically difficult to quantify, outcome measurements performed using laparoscopic box training tools under standardized conditions might offer a feasible approach. To date, the effect of music exposure at different SPLs on the training performance of laparoscopic novices has not been evaluated systematically under standardized conditions. Therefore, we chose a highly standardized stetting for this study in order to maintain the ability to transfer the findings to a real-life OR setting. Simulation-based training in minimally invasive surgery has been validated for the “Luebecker Toolbox” [13]. Transferability of the task content to a (sub)-realistic setting could be demonstrated [14]. Nonetheless, besides training, individual talent also constitutes an important factor in mastering laparoscopic skills [33]. The influence of SPL on laparoscopic tasks has not been evaluated yet, although a positive impact on accuracy has already been shown for relaxing auditory influences, such as classical music on laparoscopic tasks [34]. Our data are in line with these preliminary data that background music at a moderate SPL of 70 dB has a positive effect on performance in comparison to higher or lower SPL, although the highest total relative improvement in all exercises was within the 60 dB group. In this context, it might be relevant that most participants did not feel distracted by the music in our study. In contrast to a real-world setting within an OR there were no other pressuring factors that might have influenced performance. This fits to the results that overall course satisfaction was very high.

### Limitations

Our study design shows several potential limitations. Although a high standardization in the study design was intended, performance outcome of surgical techniques (such as laparoscopy) is methodologically difficult to quantify. Studies have shown that it is difficult to predict baseline laparoscopic surgery skills [35]. Moreover, our findings could have been relevantly biased due to differing subjective music perceptions, i.e., some students probably liked the music being played better than others, with a varying effect on their performance (“arousal-and-mood-hypothesis”) [36]. Studies have also shown that a listener’s fondness of the music being played influences their performance [37]. Furthermore, we did not use virtual reality simulators and therefore were not able to track the movement of the probands. Thus, the accuracy factor as part of the overall performance could not be recorded accordingly. In addition, the cohort size was relatively small; however, it could still deliver significant results.

In addition, the implication of transferring study results from a simulator to the OR has not been clarified yet, although it is likely that the skills themselves can be transferred [38–40]. Further analyses might focus on other factors that might influence the performance of standardized laparoscopic tasks, e.g., differing music genres.

## Conclusions

In general, along with previous studies, we could show that there is no negative effect of background music being played while performing exercises on a trainer in a standardized stetting. Moreover, our study suggests that even with rising sound pressure levels, performance is better than in a control group or a noise-shielded group. Here, the effect of blocking out music while performing the exercises might become relevant. It can be assumed that background music at a specific SPL even might enhance performance more than turning off music rigorously.

To our knowledge, our prospective trial is the first study to systematically examine the influence of different sound pressure levels on laparoscopic performance of medical novices. Future trials need to show the influence of other distractors in the operating room, such as talking or answering phone calls. Moreover, it is still not known whether the music genre makes a difference for outcome performance.

## Data Availability

All data are available upon reasonable request.
